# Effect of renal sympathetic nerve on adrenergically and angiotensin II-induced renal vasoconstriction in normal Wistar-Kyoto rats

**DOI:** 10.3109/03009734.2010.526723

**Published:** 2011-02-11

**Authors:** Mohammed H. Abdulla, Munavvar A. Sattar, Nor A. Abdullah, Abdul Hye Khan, Kolla R. L. Anand Swarup, Hassaan A. Rathore, Raisa N. Kazi, Fathihah Basri, Edward J. Johns

**Affiliations:** ^1^School of Pharmaceutical Sciences, Universiti Sains Malaysia, Minden, PenangMalaysia; ^2^Department of Pharmacology, Faculty of Medicine, Universiti Malaya, Kuala LumpurMalaysia; ^3^Cardiovascular Research Center, Medical College of Wisconsin, MilwaukeeUSA; ^4^Department of Physiology, Aras Windle, University College Cork, College Road, CorkIreland

**Keywords:** α_1_-Adrenoceptors, carvedilol, losartan, renal haemodynamics, Wistar-Kyoto rats

## Abstract

**Background:**

This study examined the effect of renal sympathetic innervation on adrenergically and angiotensin II (Ang II)-induced renal vasoconstriction in Wistar-Kyoto (WKY) rats.

**Methods:**

Forty-eight WKY rats were treated with either losartan (10 mg/kg/day p.o.) or carvedilol (5 mg/kg/day p.o.) or a combination of them (10 mg/kg/day + 5 mg/kg/day p.o.) for 7 days. On day 8, the rats were anaesthetized, and renal vasoconstrictor experiments were carried out. A group of rats was subjected to acute unilateral renal denervation during the acute study. Changes in the renal vasoconstrictor responses were determined in terms of reductions in renal blood flow caused by Ang II, noradrenaline (NA), and methoxamine (ME).

**Results:**

In normal animals, losartan decreased (*P* < 0.05) the renal vasoconstrictor response to Ang II but not to NA or ME. Carvedilol treatment, however, blunted (*P* < 0.05) the renal vasoconstrictor responses to Ang II and adrenergic agonists. Combination of losartan and carvedilol blunted (*P* < 0.05) the renal vasoconstrictor response to Ang II but augmented the responses to NA and ME (all *P* < 0.05). Interestingly, when denervated rats were treated with the same combination, there was a reduction (*P* < 0.05) in the renal vasoconstrictor responses to Ang II and adrenergic agonists.

**Conclusions:**

Data suggest that the renal sympathetic nerve contributes to adrenergic agonist-mediated renal vasoconstrictions in normal rats. The data further indicate an interactive relationship between renin-angiotensin and sympathetic nervous systems in modulating adrenergically and Ang II-induced renal vasoconstriction in WKY rats.

## Introduction

Angiotensin II (Ang II) has been shown to increase vascular sensitivity to noradrenaline in rats as well as in isolated vessels (1), hence suggesting that Ang II and noradrenaline exert synergistic actions on vasculature. It has also been reported that the blockade of endogenous Ang II by angiotensin II receptor type 1 (AT_1_) blockers could alter vascular reactivity to exogenous noradrenaline (2).

One of the most important sites of physiological action of Ang II is the renal vasculature, on which it has a direct and potent vasoconstrictor action (3). It has been reported that a certain degree of renin-angiotensin system (RAS) activity is necessary to optimize the release of noradrenaline from renal sympathetic nerve terminals (4). It has also been mentioned that noradrenaline released from renal sympathetic nerves initiates renin release by stimulation of β_1_-adrenoceptors on renin-containing juxtaglomerular granular cells (5). The renin released subsequently forms Ang II that exerts pre- and postsynaptic action on Ang II receptors (4). As reported in dog, dependence of the renal vasoconstrictor response to Ang II on an intact renal innervation, indeed, suggested a role of this peptide in catecholamine release, and it is believed that the renal vasculature is particularly sensitive to the release of catecholamine through an action of Ang II (3). Moreover, there is evidence that the elevated vascular noradrenaline sensitivity by Ang II may be attributed to a decline in catecholamine clearance because it has been reported that Ang II diminishes neuronal catecholamine uptake (6).

In the present study, an effort has been made to gain insight into the role of renal nerves on the renal vascular responsiveness to Ang II and adrenergic agonists in normotensive Wistar-Kyoto (WKY) rats. The renal vascular tone and responsiveness were assessed under basal conditions and in the presence of acute stimulation with noradrenaline (NA), methoxamine (ME), and Ang II that were infused directly into the renal artery. Series of experiments were carried out in rats treated with or without RAS and sympathetic nervous system (SNS) blockers. The blockade was caused either by pharmacological or surgical procedures, and the influence of these blockades was assessed in terms of their role in modulating adrenergically and Ang II-induced renal vasoconstriction. Therefore, the hypothesis tested in this study was that the renal haemodynamic effects of Ang II and adrenergic agonists are influenced by the presence of an intact renal innervation.

## Methods

### Animals

Experiments were conducted on age-matched male WKY rats (*n* = 48) collected from Animal Care Facility, Universiti Sains Malaysia, Penang, Malaysia. Animal handling and all procedures on animals were carried out in accordance with the guidelines of the Animal Ethics Committee, Universiti Sains Malaysia, Penang, Malaysia and had their approval. The animals were fed standard rat chow with free access to tap water and kept on a 12-h light:12-h dark cycle. Following a week of acclimatization, animals were randomly assigned into eight groups, namely control (CT), denervated (DNX), losartan (L), denervated losartan (DNX-L), carvedilol (CV), denervated carvedilol (DNX-CV), losartan+carvedilol (LCV), and denervated (losartan+carvedilol) treated (DNX-LCV) WKY rats (all *n* = 6). Rats treated with carvedilol (Dilatrend, Roche, Basel, Switzerland) received the drug at a dose of 5 mg/kg/day (p.o.) for 7 days (7–9). Losartan (Cozaar, MSD, NJ, USA) was given at an oral dose of 10 mg/kg/day as previously mentioned (10–13). A group of rats received losartan (10 mg/kg/day, p.o.) along with carvedilol (5 mg/kg/day, p.o.) (7,11). Following the 7-day treatment period, the overnight fasted animals were subjected to renal vasoconstrictor study.

### Haemodynamic studies

Surgical procedures on rats were similar to those described previously (14–16) with some modifications. The overnight (10–12 h) fasted rats were anaesthetized with 60 mg/kg (i.p.) sodium pentobarbitone (Nembutal, CEVA, Libourne, France). The trachea was cannulated to facilitate respiration. The right carotid artery was catheterized (PP50, Portex, Kent, UK) and connected to a fluid-filled pressure transducer (P23 ID Gould, Statham Instruments, Nottingham, UK) linked to a computerized data acquisition system (PowerLab, ADInstruments, Sydney, Australia) for continuous monitoring of mean arterial blood pressure (MAP) and heart rate (HR) throughout the experiment. The left jugular vein was cannulated to infuse maintenance dose of anaesthetic whenever needed. Subsequently, via a ventral mid-line incision, the left kidney was exposed followed by cannulation of the left iliac artery (PP50, Portex, UK) for continuous infusion of normal saline at 6 mL/h throughout the experiment. The iliac artery cannula was advanced through the abdominal aorta until its tapered tip faced the origin of the left renal artery to allow optimum administration of drugs into the renal artery (11,15–17). The renal artery was cleared to allow placing of an electromagnetic flow probe (EP 100 series, Carolina Medical Instruments, King, NC, USA). The probe was connected to a square-wave electromagnetic flow-meter (Carolina Medical Instruments, King, NC, USA) which was further linked to a computerized data acquisition system (PowerLab, ADInstruments, Sydney, Australia). Upon completion of the surgery, the animal was stabilized for an hour before starting the acute renal vasoconstrictor experiment.

### Acute renal denervation

Acute unilateral renal denervation of the left kidney was performed according to previous studies (7,12,13). The renal artery was carefully stripped off from the surrounding covering tissues followed by cutting of the renal nerve and coating the remaining tissue with 10% phenol in absolute alcohol (7,18,19). The denervation was tested by placing a silver wire electrode to the coeliac ganglia at which electrical stimulation (15 V, 1–4 Hz, 0.2 ms) was applied from a stimulator for 10 s. If there was a significant fall in renal blood flow (RBF), more dissection and cutting of renal nerves were done until no change in the RBF was observed during stimulation (18,19).

### Experimental protocol

Acute renal vasoconstrictor experimental protocol involved the administration of vasoactive agents that led to constriction of the renal artery, hence reduction in the RBF. Bolus doses of noradrenaline (NA) (Sanofi Winthrop, Surry, UK) (25, 50, 100, and 200 ng), methoxamine (ME) (Wellcome, London, UK) (0.5, 1, 2, and 4 μg), and angiotensin II (Ang II) (CIBA-GEIGY, Basel, Switzerland) (2.5, 5, 10, and 20 ng) were injected in ascending and descending orders into the intra-renal infusion line in order to ensure their delivery into the renal artery. A series of time control experiments were carried out to examine any time-dependent changes in the experimental protocol. In this series of experiments, MAP and RBF were measured in a way that matched exactly the protocol which is used with drug administration.

The intra-renal administration of agonists was carried out very carefully with a goal of producing renal effect without causing any significant change in the systemic blood pressure. A 10-min interval was allowed for recovery after each agonist treatment phase. MAP and RBF were monitored continuously throughout the experiment; however, only the values that were measured at the beginning of the renal vasoconstrictor experiment with each of the agonists (NA, ME, and Ang II) were considered as the basal MAP and RBF values for each of the corresponding agonists.

### Statistical analysis and presentation of data

The vasoconstrictor responses caused by Ang II and adrenergic agonists were taken as the average values caused by each dose of the agonist administered and applied in ascending and descending orders. The overall mean response for each agonist was taken as the average value of the vasoconstrictor responses (drop in RBF) obtained at each dose. The data on the drop of RBF were expressed as the percentage. Drop in relation to the baseline value. All data were expressed as mean % reduction ± SEM of renal vasoconstrictor responses elicited by all the doses of Ang II, NA, and ME, and compared between CT, DNX, L, DNX-L, CV, DNX-CV, LCV, and DNX-LCV-treated WKY rats. Data were analysed by either two- or one-way ANOVA followed by Bonferroni *post-hoc* test. The differences between the means were considered significant at 5% level.

## Results

**Figure 1.**
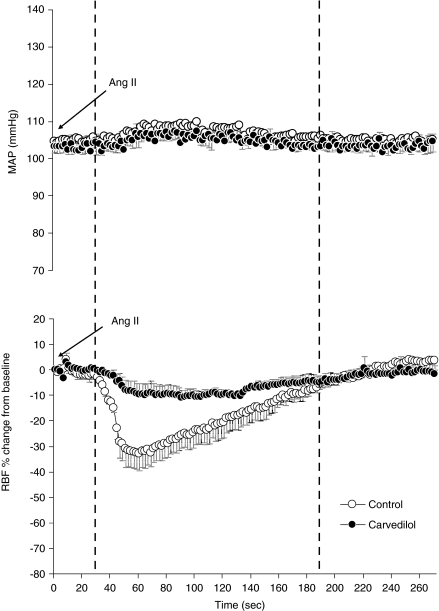
Time course of the renal vasoconstrictor response to Ang II. MAP changes (top) and RBF response (bottom) following the administration of a bolus injection (20 ng) into the renal artery. The delay of about 30 s at the beginning of the response is due to the travel time in the cannula before reaching the renal vasculature. Stippled lines denote ± SEM, *n* = 5 rats.

The base-line MAP and RBF as well as renal vascular resistance (RVR) or HR in DNX, L, DNX-L, CV, DNX-CV, LCV, and DNX-LCV-treated rats were not different from CT (Table I). The MAP recorded before and after administration of the agonists was similar, and this trend was observed throughout the experiment. As an example, Figure 1 illustrates this phenomenon and shows the MAP and RBF responses to the injection of the highest dose of Ang II (20 ng) in rats either treated or untreated with carvedilol.

**Table I.** Base-line values of body weight, mean arterial blood pressure (MAP), renal blood flow (RBF), renal vascular resistance (RVR), and heart rate (HR) in CT, DNX, L, DNX-L, CV, DNX-CV, LCV, and DNX-LCV. All data are expressed as mean ± SEM.

Treatment	*n*	Body weight (g)	MAP (mmHg)	RBF (mL min^-1^ kg^-1^)	RVR (mmHg min kg mL^-1^)	HR (bpm)
CT	6	277 ± 8	110 ± 3	9.0 ± 1.0	13.4 ± 2.1	285 ± 12
DNX	6	281 ± 10	109 ± 7	10.1 ± 2.6	11.4 ± 2.2	312 ± 17
L	6	270 ± 7	110 ± 10	8.6 ± 3.1	8.9 ± 0.1	301 ± 13
DNX-L	6	278 ± 8	109 ± 9	11.8 ± 1.5	14.3 ± 4.1	299 ± 14
CV	6	288 ± 9	111 ± 4	11.6 ± 4.9	11.6 ± 4.6	258 ± 19
DNX-CV	6	290 ± 9	108 ± 8	9.8 ± 3.2	10.9 ± 3.1	300 ± 20
LCV	6	272 ± 5	115 ± 6	10.6 ± 2.5	11.4 ± 2.1	258 ± 19
DNX-LCV	6	284 ± 8	118 ± 12	11.2 ± 1.2	11.2 ± 0.7	237 ± 11

bpm = beats per minute.

### Renal vasoconstrictor responses

#### Noradrenaline (NA)

**Figure 2.**
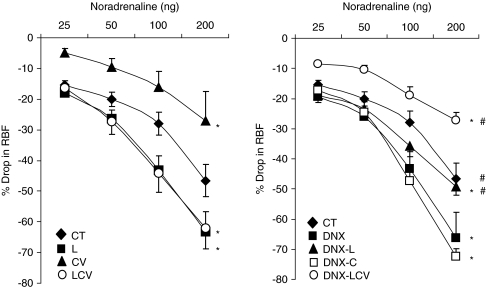
Renal vasoconstrictor responses to graded doses of NA in CT, DNX, L, DNX-L, CV, DNX-CV, LCV, and DNX-LCV. *P < 0.05 compared to CT. ^#^*P* < 0.05 compared to DNX. Data were analysed by two-way ANOVA followed by Bonferroni *post-hoc* test, *n* = 6 rats.

Noradrenaline caused a dose-dependent reduction in the RBF. The renal vascular response to NA was smaller (*P* < 0.05) in CV-treated rats as compared to CT (CV 14.2% ± 3.0% versus CT 32.2% ± 3.0%). Quite in contrast, there was no change in the renal vasoconstrictor response to NA in L or DNX-L-treated rats compared to CT (L 34.4% ± 3.5% and DNX-L 32.7% ± 2.4% versus CT 32.2% ± 3.0%). In rats treated with a combination of losartan and carvedilol (LCV), the reduction in RBF in response to NA was greater than CT (LCV 37.3% ± 3.7% versus CT 32.2% ± 3.0%; *P* < 0.05). DNX-LCV rats had a markedly lower (*P* < 0.05) renal response to NA compared to CT (DNX-LCV 16.2% ± 1.5% versus CT 32.2% ± 3.0%) (Figure 2). Finally, CT, DNX-L, and DNX-LCV rats had lower overall mean renal vasoconstrictor responses to NA compared to DNX (CT 32.2% ± 3.0%, DNX-L 32.7% ± 2.4%, and DNX-LCV 16.2% ± 1.5% versus DNX 39.0% ± 4.0%; all *P* < 0.05) (Figure 2).

#### Methoxamine (ME)

**Figure 3.**
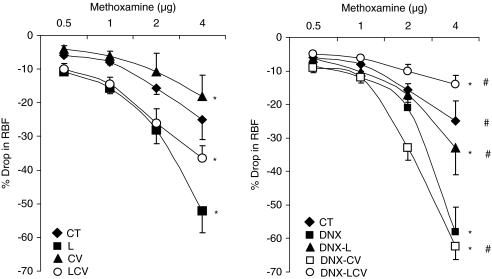
Renal vasoconstrictor responses to graded doses of ME in CT, DNX, L, DNX-L, CV, DNX-CV, LCV, and DNX-LCV. *P < 0.05 compared to CT. ^#^*P* < 0.05 compared to DNX. Data were analysed by two-way ANOVA followed by Bonferroni *post-hoc* test, *n* = 6 rats.

Intra-renal arterial bolus doses of this agonist resulted in dose-dependent reductions in RBF (Figure 3). The magnitude of RBF reduction in CV was lower than CT (CV 9.6% ± 2.4% versus CT 15.7% ± 4.1%; *P* < 0.05). There was, however, no difference in the renal response to ME between L or DNX-L, and CT (L versus CT: 19.5% ± 3.4% versus 15.7% ± 4.1%, and DNX-L versus CT: 17.5% ± 3.1% versus 15.7% ± 4.1%). The vasoconstrictor responses to ME in LCV were greater than CT (LCV versus CT: 22.0% ± 2.2% versus 15.7% ± 4.1%; *P* < 0.05). The renal responses to ME in DNX-LCV, however, were significantly lower than CT (DNX-LCV versus CT: 8.7% ± 1.0% versus 15.7% ± 4.1%; *P* < 0.05) (Figure 3). Further, the vasoconstrictor response to ME in CT, DNX-L, and DNX-LCV were lower than DNX (CT versus DNX: 15.7% ± 4.1% versus 24.7% ± 3.5%, DNX-L versus CT: 17.5% ± 3.1% versus 24.7% ± 3.5%, and DNX-LCV versus DNX: 8.7% ± 1.0% versus 24.7% ± 3%; all *P* < 0.05) (Figure 3).

#### Angiotensin II (Ang II)

**Figure 4.**
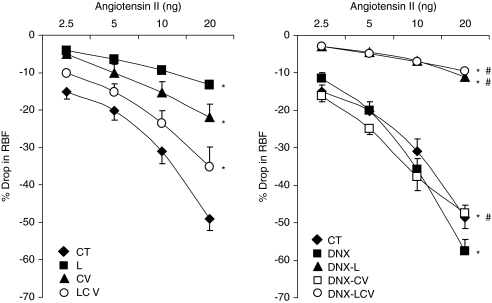
Renal vasoconstrictor responses to graded doses of Ang II in CT, DNX, L, DNX-L, CV, DNX-CV, LCV, and DNX-LCV. *P < 0.05 compared to CT. ^#^*P* < 0.05 compared to DNX. Data were analysed by two-way ANOVA followed by Bonferroni *post-hoc* test, *n* = 6 rats.

Ang II also caused a dose-related reduction in RBF (Figure 4). It was found that the level of renal vasoconstriction as assessed from the reduction of RBF was lower in L, DNX-L, and CV compared to CT (L versus CT: 8.1% ± 0.7% versus 28.5% ± 2.3%, DNX-L versus CT: 6.7% ± 0.7% versus 28.5% ± 2.3%, CV versus CT: 12.4% ± 1.6% versus 28.5% ± 2.3%; all *P* < 0.05). A similar trend was also observed in the LCV and DNX-LCV groups compared to CT (LCV versus CT: 21.3% ± 2.1% versus 28.5% ± 2.3%, and DNX-LCV versus CT: 6.1% ± 2.1% versus 28.5% ± 2.3%; all *P* < 0.05) (Figure 4). Finally, DNX-L and DNX-LCV rats had lower (*P* < 0.05) responses to Ang II compared to DNX (DNX-L versus DNX: 6.7% ± 0.7% versus 31% ± 2.9%, and DNX-LCV versus DNX: 6.1% ± 2.1% versus 31% ± 2.9%; all *P* < 0.05) (Figure 4).

## Discussion

This study investigated the role of the renal sympathetic nervous system on Ang II and adrenergic agonist-induced vasoconstrictor responses in the renal vasculature of WKY rats. This was done by studying the modulation of adrenergically and Ang II-induced renal vasoconstrictions in the presence or absence of a blockade of RAS, SNS, or both. The AT_1_-receptor blocker losartan was used to block RAS, while carvedilol treatment as well as surgical denervation were used to block the SNS. As reported earlier, the dose of losartan used in this study has been shown to be sufficient to inhibit Ang II action on AT_1_-receptors (11). The dose of carvedilol used in this study was based on earlier reports from others (8,9) and from our previous study, where carvedilol markedly blunted phenylephrine-mediated vasoconstrictor response (11).

In the present study, we found that in WKY rats blocking of the sympathetic innervations to the kidney augmented the renal responses to NA and ME, possibly due to enhancement of the receptors sensitivity to the given agonists (7,12,20), but it did not cause any significant change in the vasoconstrictor response to Ang II. It is suggested that the released noradrenaline in neurons interacts with α_1_-adrenoceptors, and their chronic activation in turn results in the down-regulation of AT_1_-receptors (21). With this finding, it appears that in the absence of endogenous noradrenaline the postsynaptic AT_1_-receptors in the renal vasculature of the WKY rats were not affected and, therefore, mediated the vasoconstrictor action of Ang II. The unaffected Ang II-mediated renal vasoconstriction in the absence of renal nerve can be further explained by an earlier finding that Ang II per se facilitates sympathetic neurotransmission via prejunctionally located AT_1_-receptors (22). These presynaptic effects of Ang II are found in renal vasculature (23,24). These observations led us to suggest that in the renal vasculature there is, indeed, a relationship between AT_1_ and adrenoceptors in mediating actions of Ang II and adrenergic stimuli.

In the present study it was also observed that the blocking of adrenergic receptors by carvedilol blunted the renal vasoconstrictor action of both Ang II and adrenergic agonists. Ang II has been shown to influence the expression of α_1_-adrenoceptors in rat vascular smooth muscle (25) and, hence, suggests that indirect effects of Ang II could be mediated in part by increased expression of α_1_-adrenoceptors (25). Although studying the expression of receptors in the renal vasculature was beyond the scope of this study, based on the changes observed in renal blood flow we suggest that in WKY rats blocking of α_1_-adrenoceptors perhaps resulted in the reduction of AT_1_-receptors sensitivity to exogenous Ang II. This observation further indicated a possible interdependency in the actions of adrenergic stimuli and Ang II in vascular smooth muscle and strengthens our view of an interactive relationship between AT_1_ and adrenoceptors in the renal vasculature. Collectively, these observations indicated an interactive relationship between RAS and SNS in these rats, and we have earlier reported such interactive relationship between these two systems in a rat model of essential hypertension, spontaneously hypertensive rat (SHR) (7).

There was a marked attenuation in the renal vasoconstrictor responses to Ang II in normal rats treated with losartan. However, there was no change in the renal vasoconstrictor responses to NA and ME, and this indicates a weak influence of the blockade of the postsynaptic AT_1_-receptors on α_1_-adrenoceptor-mediated renal vascular responses in normal WKY rats. This finding was in accord with an earlier report demonstrating that losartan dose-dependently decreased the renal vasoconstriction response to renal sympathetic nerve stimulation but not to exogenously administered noradrenaline (4).

This study showed that carvedilol treatment attenuated the renal vasoconstrictions induced by intra-renally administered Ang II. Indeed, this observation indicated an adrenergic influence to the renal response to Ang II in WKY rats and is in agreement with an earlier study in anaesthetized rabbits (3). In line with these observations, we suggest an interactive relationship between postsynaptic α_1_-adrenoceptors and AT_1_-receptors in the renal vasculature of WKY rat, as blocking of the former by carvedilol influenced the sensitivity of the latter to Ang II. Our suggestion of a possible interactive relationship between RAS and SNS is further supported by an earlier study in human that suggested a cross-talk between AT_1_- and α_1_-adrenoceptors, and also showed that in the presence of an increased sympathetic tone carvedilol provides AT_1_-receptor blockade via its α_1_-adrenoceptor-blocking effects (26).

In conclusion, this study showed that there is an interactive relationship between AT_1_ and α_1_-adrenoceptors in modulating renal haemodynamic responses to Ang II and adrenergic agonists in WKY rats. Further, we found that in WKY rats this relationship between AT_1_ and α_1_-adrenoceptors is influenced by the renal nerve.

## References

[1] Marano G, Argiolas L (1994). Postjunctional regulation by angiotensin II of alpha 1-adrenoceptor-mediated pressor responses in the rat. Eur J Pharmacol.

[2] Raasch W, Dominiak P, Ziegler A, Dendorfer A (2004). Reduction of vascular noradrenaline sensitivity by AT1 antagonists depends on functional sympathetic innervation. Hypertension.

[3] Chen K, Zimmerman BG (1995). Angiotensin II-mediated renal vasoconstriction amenable to alpha 1-adrenoceptor blockade. Eur J Pharmacol.

[4] DiBona GF (2000). Nervous kidney. Interaction between renal sympathetic nerves and the renin-angiotensin system in the control of renal function. Hypertension.

[5] Holmer SR, Kaissling B, Putnik K, Pfeifer M, Kramer BK, Riegger GA (1997). Beta-adrenergic stimulation of renin expression in vivo. J Hypertens.

[6] Vatta MS, Bianciotti LG, Locatelli AS, Papouchado ML, Fernandez BE (1992). Monophasic and biphasic effects of angiotensin II and III on norepinephrine uptake and release in rat adrenal medulla. Can J Physiol Pharmacol.

[7] Abdulla MH, Sattar MA, Khan MA, Abdullah NA, Johns EJ (2009). Influence of sympathetic and AT-receptor blockade on angiotensin II and adrenergic agonist-induced renal vasoconstrictions in spontaneously hypertensive rats. Acta Physiol (Oxf).

[8] Rodriguez Perez JC, Cabrera JJ, Anabitarte A, Plaza ML, Losada A, Garcia Suarez P (2001). [Effects of carvedilol in rats with induced chronic kidney failure]. Nefrologia.

[9] Rodriguez-Perez JC, Losada A, Anabitarte A, Cabrera J, Llobet J, Palop L (1997). Effects of the novel multiple-action agent carvedilol on severe nephrosclerosis in renal ablated rats. J Pharmacol Exp Ther.

[10] Bayorh MA, Ganafa AA, Eatman D, Walton M, Feuerstein GZ (2005). Simvastatin and losartan enhance nitric oxide and reduce oxidative stress in salt-induced hypertension. Am J Hypertens.

[11] Abdulla MH, Sattar MA, Abdullah NA, Khan MA, Abdallah HH, Johns EJ (2009). Chronic treatment with losartan and carvedilol differentially modulates renal vascular responses to sympathomimetics compared to treatment with individual agents in normal Wistar Kyoto and spontaneously hypertensive rats. Eur J Pharmacol.

[12] Abdulla MH, Sattar MA, Salman IM, Abdullah NA, Ameer OZ, Khan MA (2008). Effect of acute unilateral renal denervation on renal hemodynamics in spontaneously hypertensive rats. Auton Autacoid Pharmacol.

[13] Abdulla MH, Sattar MA, Abdullah NA, Hazim AI, Anand Swarup KR, Rathore HA (2008). Inhibition of Ang II and renal sympathetic nerve influence dopamine- and isoprenaline-induced renal haemodynamic changes in normal Wistar-Kyoto and spontaneously hypertensive rats. Auton Autacoid Pharmacol.

[14] Hye Khan MA, Sattar MA, Abdullah NA, Johns EJ (2008). Influence of combined hypertension and renal failure on functional alpha(1)-adrenoceptor subtypes in the rat kidney. Br J Pharmacol.

[15] Just A, Olson AJ, Whitten CL, Arendshorst WJ (2007). Superoxide mediates acute renal vasoconstriction produced by angiotensin II and catecholamines by a mechanism independent of nitric oxide. Am J Physiol Heart Circ Physiol.

[16] Armenia A, Sattar MA, Abdullah NA, Khan MA, Johns EJ (2008). Functional subtypes of renal alpha1-adrenoceptor in diabetic and non-diabetic 2K1C Goldblatt renovascular hypertension. Acta Pharmacol Sin.

[17] Khan MA, Sattar MA, Abdullah NA, Johns EJ (2008). Alpha1B-adrenoceptors mediate adrenergically-induced renal vasoconstrictions in rats with renal impairment. Acta Pharmacol Sin.

[18] Takishita S, Muratani H, Sesoko S, Teruya H, Tozawa M, Fukiyama K (1994). Short-term effects of angiotensin II blockade on renal blood flow and sympathetic activity in awake rats. Hypertension.

[19] Rademacher R, Berecek KH, Ploth DW (1986). Effects of angiotensin inhibition and renal denervation in two-kidney, one clip hypertensive rats. Hypertension.

[20] Cleary L, Slattery J, Bexis S, Docherty JR (2004). Sympathectomy reveals alpha 1A- and alpha 1D-adrenoceptor components to contractions to noradrenaline in rat vas deferens. Br J Pharmacol.

[21] Sumners C, Raizada MK, Kang J, Lu D, Posner P (1994). Receptor-mediated effects of angiotensin II on neurons. Front Neuroendocrinol.

[22] Balt JC, Mathy MJ, Pfaffendorf M, van Zwieten PA (2003). Sympatho-inhibitory actions of irbesartan in pithed spontaneously hypertensive and Wistar-Kyoto rats. Fundam Clin Pharmacol.

[23] Wong PC, Hart SD, Timmermans PB (1991). Effect of angiotensin II antagonism on canine renal sympathetic nerve function. Hypertension.

[24] Wong PC, Bernard R, Timmermans PB (1992). Effect of blocking angiotensin II receptor subtype on rat sympathetic nerve function. Hypertension.

[25] Hu ZW, Shi XY, Okazaki M, Hoffman BB (1995). Angiotensin II induces transcription and expression of alpha 1-adrenergic receptors in vascular smooth muscle cells. Am J Physiol.

[26] Batenburg WW, van Esch JH, Garrelds IM, Jorde U, Lamers JM, Dekkers DH (2006). Carvedilol-induced antagonism of angiotensin II: a matter of alpha1-adrenoceptor blockade. J Hypertens.

